# Soporte circulatorio mecánico en choque cardiogénico, una realidad en el Perú

**DOI:** 10.47487/apcyccv.v1i3.84

**Published:** 2020-09-30

**Authors:** Leonardo Salazar

**Affiliations:** 1 Chair ELSO LATAM. Chair ELSO LATAM; Fundación Cardiovascular de Colombia Fundación Cardiovascular Colombia

El choque cardiogénico es una condición compleja y heterogénea con alta mortalidad y morbilidad. Se caracteriza por un estado de hipoperfusión celular originado en una alteración de la fisiología hemodinámica. Esta hipoperfusión conduce a grados variables de disfunción orgánica con aumento de la fragilidad del paciente que lo lleva con frecuencia a la muerte. El abordaje diagnóstico y terapéutico del choque cardiogénico es desafiante por múltiples motivos; diversas enfermedades de orígenes biológicos muy distintos pueden desarrollar esta condición y solo pocas pueden resolverse. Frecuentemente se dispone de poco tiempo para detener y revertir la hipoperfusión celular. El abordaje terapéutico depende de las causas específicas de la alteración hemodinámica, y cuando no es posible revertir o compensar estas alteraciones se produce un choque refractario que rápidamente se vuelve irreversible ^1^.

El soporte circulatorio mecánico aparece como una estrategia para recuperar la perfusión celular cuando la alteración hemodinámica es refractaria. Existen diversas estrategias de soporte circulatorio mecánico; las más utilizadas en Latinoamérica son la oxigenación con membrana extracorpórea venoarterial (ECMO VA) y la asistencia ventricular temporal. El ECMO VA se puede implementar de manera rápida, percutánea y en la cama del paciente; provee un soporte hemodinámico y respiratorio completo, aunque con un potencial de lesión cardiaca y pulmonar. La asistencia ventricular temporal requiere de una intervención quirúrgica y es menos versátil que el ECMO VA; sin embargo, este tipo de soporte disminuye eficientemente la carga y la presión ventricular disminuyendo también la congestión pulmonar. A pesar de ser herramientas poderosas para mejorar el gasto cardiaco su uso en choque cardiogénico aún se acompaña de mortalidades muy altas ^2^.

Implementar de manera exitosa el soporte circulatorio mecánico es objeto de estudio y debate intenso en la comunidad académica. Aún no hay respuestas infalibles sobre cuándo es el momento oportuno de indicar estas terapias, o de cómo se disminuyen sus complicaciones; de cuándo pasar de un tipo de soporte a otro y de cómo se define la terapia final de un paciente que no recupera la función cardiaca ^3^. Integrar el soporte circulatorio mecánico en un programa de cardiología y cirugía cardiovascular es un desafío que requiere un apoyo político decidido de la dirección y un nivel de integración y trabajo interdisciplinario muy superior. Esta empresa se acompaña comúnmente de frustraciones y conflictos por lo que se requiere perseverancia y claridad en los propósitos para avanzar y consolidar el equipo de soporte circulatorio mecánico.

Muchos opinan que una tarea tan compleja y costosa no debería intentarse en los países en vías de desarrollo; sin embargo, permitir que sean nuestros medios y no nuestras esperanzas los que definan nuestros propósitos es condenarnos al subdesarrollo para siempre. Afortunadamente varios centros en diversos países latinoamericanos han desafiado este paradigma. El remplazo de la función cardiaca con dispositivos de corto y largo plazo, y el trasplante cardiaco salvan muchas vidas en Perú, Brasil, Argentina, Chile, Uruguay, Paraguay, Colombia, Panamá, Costa Rica y México.

Por las condiciones de desigualdad e inequidad en Latinoamérica estos proyectos se han desarrollado, principalmente, en hospitales privados. En el Perú esto no ha sido así. El desarrollo del trasplante cardiaco y el soporte circulatorio mecánico ha ocurrido inicialmente en hospitales públicos. El grupo de ECMO del Instituto Nacional Cardiovascular (ver artículo de Pitta *et al.*, Arch Peru Cardiol Cir Cardiovasc. 2020;1(3):164-169), reporta sus resultados en un grupo de pacientes con síndrome coronario agudo y choque cardiogénico refractario, que requirieron manejo con ECMO VA y asistencia ventricular temporal. Este escenario conduce normalmente a la muerte, pero gracias al trabajo multidisciplinario reportan una sobrevida mayor al 40% integrando la asistencia ventricular cuando fuera necesario. He tenido el privilegio de conocer el programa del Instituto Nacional Cardiovascular desde sus inicios y el profesionalismo y la convicción de sus médicos especialistas, enfermeras y terapeutas es excepcional. Nos demuestran que la voluntad y el trabajo en equipo son capaces de superar todos los obstáculos.

No debemos olvidar que los verdaderos pioneros son los pacientes. Que nuestros esfuerzos son también una promesa a los que fallecieron y fallecen mientras resolvemos problemas y disminuimos la incertidumbre. Hoy, los niños y los adultos enfermos del corazón tienen mejores oportunidades gracias al esfuerzo de estos equipos multidisciplinarios que hacen parte de la gran comunidad Latinoamericana y mundial que todos los días busca soluciones para que los enfermos cardiópatas puedan vivir una vida plena.
